# Impact of Discharge Electrode Positioning on Nanoparticle Collection in a Single-Stage Wire-Plate Electrostatic Precipitator

**DOI:** 10.1021/acsomega.5c00722

**Published:** 2025-06-12

**Authors:** Raíssa G. S A Andrade, Vádila G. Guerra

**Affiliations:** Department of Chemical Engineering, Federal University of São Carlos, Rodovia Washington Luís, km 235, C.P. 676, CEP, 13560-970 São Carlos, São Paulo, Brazil

## Abstract

The electrostatic precipitator is used to collect particles and reduce their negative impacts on the environment and human health. The present work investigated the influence of the discharge electrode position on nanoparticle electrostatic precipitation. For that, experiments were performed with 1 and 3 discharge electrodes, with the inlet spacings varying from 1.5 to 28.5 cm, referring to the distance between the beginning of the collecting plate and the first discharge electrode. Under most conditions, the inlet spacings for wire positioning in the central region of the electrostatic precipitator achieved the highest collection efficiencies, because the high electric field region was in the center of the equipment duct and provided more uniform particle charging. However, high collection efficiencies were also achieved using the other inlet spacings, especially with lower air velocities and higher electric fields.

## Graphical abstract

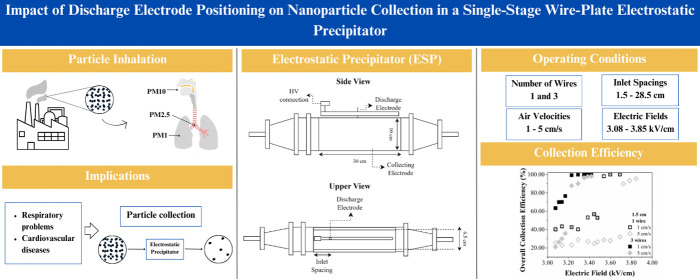

## 1 Introduction

Control of particulate air pollution is essential due to the harmful human health effects that inhalation of these particles can cause, including respiratory problems and cardiovascular diseases, in addition to the negative atmospheric effects of suspended particles.^1,2^ In humans, the risks may be greater in the case of nanoparticles (*d*_p_ < 100 nm), which can penetrate deeper into the respiratory system, as well as other organs.^3,4^ Despite their small size, these particles have a high surface area, which some studies have investigated for its potential contribution to toxicity.^3,5^

To address these problems, the electrostatic precipitator (ESP) is an excellent device for removing particles, with the advantages of low-pressure drop, low maintenance costs, and high collection efficiency.^6^ This equipment ionizes and collects particles using an electric field generated by discharge and collecting electrodes with opposite polarities.^7^ However, the ionization process can generate ozone, reducing ESP performance and limiting indoor applications.^6,8^ Higher applied voltages can increase ozone concentrations,^9^ due to the greater energy density within the ionization plasma region.^10^ Therefore, the use of lower voltages is beneficial for indoor applications.

The charging of particles occurs by two main mechanisms, namely field charging and diffusion charging. In the first, which is predominant for particles larger than 1 μm, the ions are transported by the electric field lines until they collide with particles in their trajectories.^11,12^ On the other hand, diffusion charging occurs for particles smaller than 100 nm and depends on the effect of Brownian motion, involving the probability of collision due to random movement of the particles.^7,12^ Consequently, the electric charging diminishes for nanoparticles, primarily due to the decrease in collision probability with smaller particle sizes,^13^ dropping below 5% for particles with diameters below 10 nm,^14,15^ as well as to high rates of deposition of nanoparticles on the walls of the device.^13,16^ In addition, the particle charging time is the parameter that most affects the diffusion mechanism.^8^ Therefore, it is necessary to improve the collection of particles in this diameter range. Among other options, the ESP geometry allows for different configurations that can influence the performance of the equipment, such as the electrode position. This parameter, which can affect nanoparticle collection by altering the electric field distribution inside the ESP, may be evaluated using the inlet spacing, which is the distance from the beginning of the collecting plate to the first discharge electrode.

In recent years, there has been an increase in numerical studies focused on aspects of electrostatic precipitation, in attempts to better understand the effects related to different process variables such as the charge density distribution,^17,18^ particle deposition and charging,^19–22^ electrohydrodynamic flow,^23,24^ and electric field distribution.^25–27^ According to Podliński et al.^28^ and Chun et al.,^29^ in plate-wire type electrostatic precipitators, the ionic wind strongly influences the primary flow when the electrohydrodynamic flow values are much greater than 1. This is because the ionic flow generated by the electric field disturbs the primary gas flow, with the formation of local vortices and increased turbulence near the wires. In another work, the evaluation of an electrostatic precipitator featuring collecting plates with pockets revealed that the positioning of the discharge electrodes altered both the current density distribution and the electrohydrodynamic streamlines.^26^

Several experimental studies have evaluated the effects of operating conditions, mainly considering the air velocity and the electric field,^30–34^ and geometric parameters such as the shape, number, diameter, and spacing of the collecting and discharge electrodes.^35–40^ However, no experimental study was found that evaluated the effect on nanoparticle collection of discharge electrode position, in terms of the inlet spacing. The position of the discharge electrodes can influence the electrohydrodynamics of the electrostatic precipitator, affecting particle charging and, consequently, the particle collection efficiency.

Therefore, the present work aimed to evaluate the influence of the electrode position in a single-stage wire-plate ESP. For this, an analysis was made of the performance of the ESP operating with the discharge electrode in the initial, central, and final regions of the ESP duct, to determine how the position of this component affected the charging and collection of nanoparticles.

## 2 Materials and Methods

### 2.1 Experimental Unit

The experiments were performed using the experimental unit illustrated in Figure 1. Air was supplied by a compressor (model MSV 12/175, Schulz) and passed through a purification filter (model 3074B, TSI) to remove impurities. A nanoparticle generator (model 3079, TSI) produced the aerosol from a solution of NaCl at a known concentration. This aerosol flowed through a diffusion dryer (model 3062, TSI) containing silica gel to retain excess moisture. The aerosol then passed through a krypton-85 (^85^Kr) aerosol neutralizer (model 3054, TSI), which neutralized any electrostatic charges present on the particles, to avoid their deposition on the pipe surface and effect on the electrostatic precipitation process.

**Figure 1:**
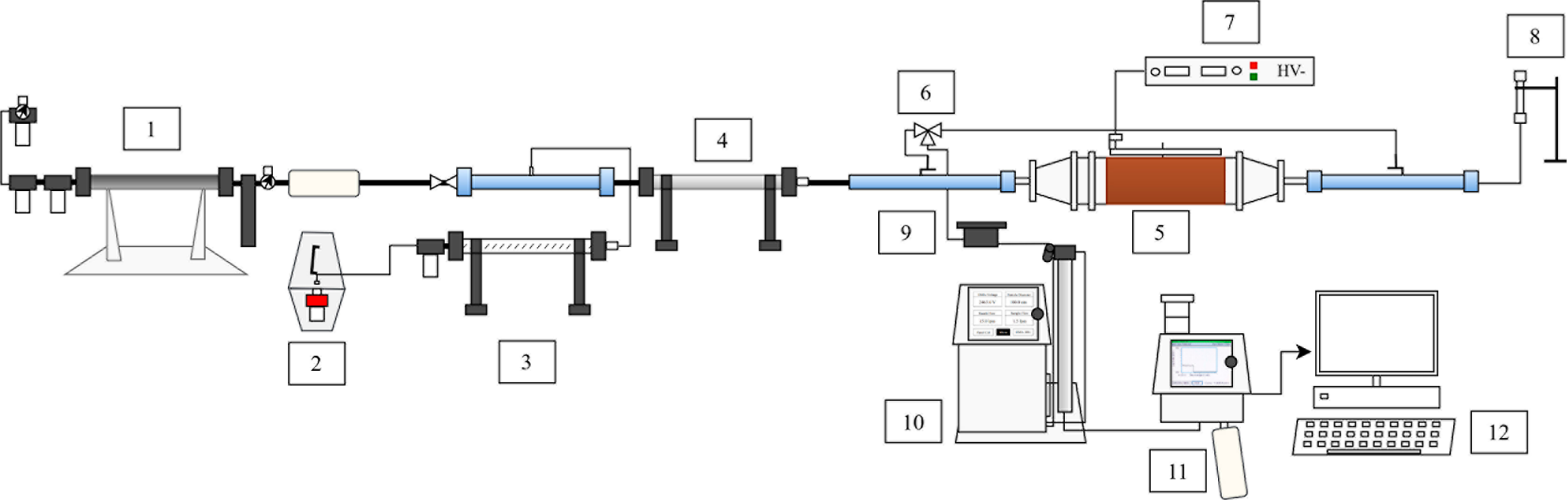
Representation of the experimental unit: (1) filtered air supply, (2) aerosol generator, (3) diffusion dryer, (4) aerosol neutralizer (Kr-85), (5) electrostatic precipitator, (6) 3-way valve, (7) high voltage power supply, (8) flowmeter, (9) aerosol neutralizer (Am-241), (10) electrostatic classifier, (11) particle counter and (12) computer.

After charge neutralization, the aerosol was dispersed in the electrostatic precipitator using a diffuser at the inlet of the equipment. A high voltage source (model SL30PN300, Spellman) supplied the voltage required to create an electric field inside the precipitator, with negative polarity, resulting in the phenomenon of chain ionization of molecules. In addition, the high voltage source also provided the electric current values within the electrostatic precipitator.

Sampling of particles to obtain the data used in subsequent efficiency calculations employed two probes with internal diameter of 2 mm and angle of 90°, installed in the piping upstream and downstream of the equipment, at distances of 26 cm from the inlet and outlet of the precipitator. The probes were fixed to the central axis of the pipe (2.6 cm internal diameter), parallel to the air flow direction and connected to a 3-way valve by rigid silicone hoses (0.4 cm internal diameter). This valve was used to switch the particle sampling point. In addition, a rotameter was installed in the electrostatic precipitator outlet piping to control the air flow.

After passing through the 3-way valve, the particles dispersed in the air passed through an americium-241 (^241^Am) aerosol neutralizer to remove the electrostatic charges acquired in the precipitator and avoid obtaining inaccurate results. Finally, the particles passed to an electrical mobility particle analysis system (SMPS) composed of an electrostatic classifier with long DMA (model 3081, TSI) and a particle counter (model 3776, TSI). This system was connected to a computer for control of the analyses and collection of data.

Further detailed information about the equipment used in the experimental unit can be found in a previous article.^35^

### 2.2 Electrostatic Precipitator and Experimental Procedure

The electrostatic precipitator used for the experiments, shown in Figure 2, was a single-stage wire-plate type, with two copper collection plates, 30 cm long and 10 cm high, spaced 6.5 cm apart. Stainless-steel discharge electrodes (smooth wires with 0.4 mm in diameter) were placed in the central region between the collector plates and the experimental tests were performed using one and three discharge electrodes. The single wire configuration was used to specifically assess the effect of the wire position in the ESP on the electrostatic precipitation of nanoparticles, while the multiwire configuration presents the effect of the number of wires and interaction between them due to potential interference from electrical shielding.^35^

**Figure 2:**
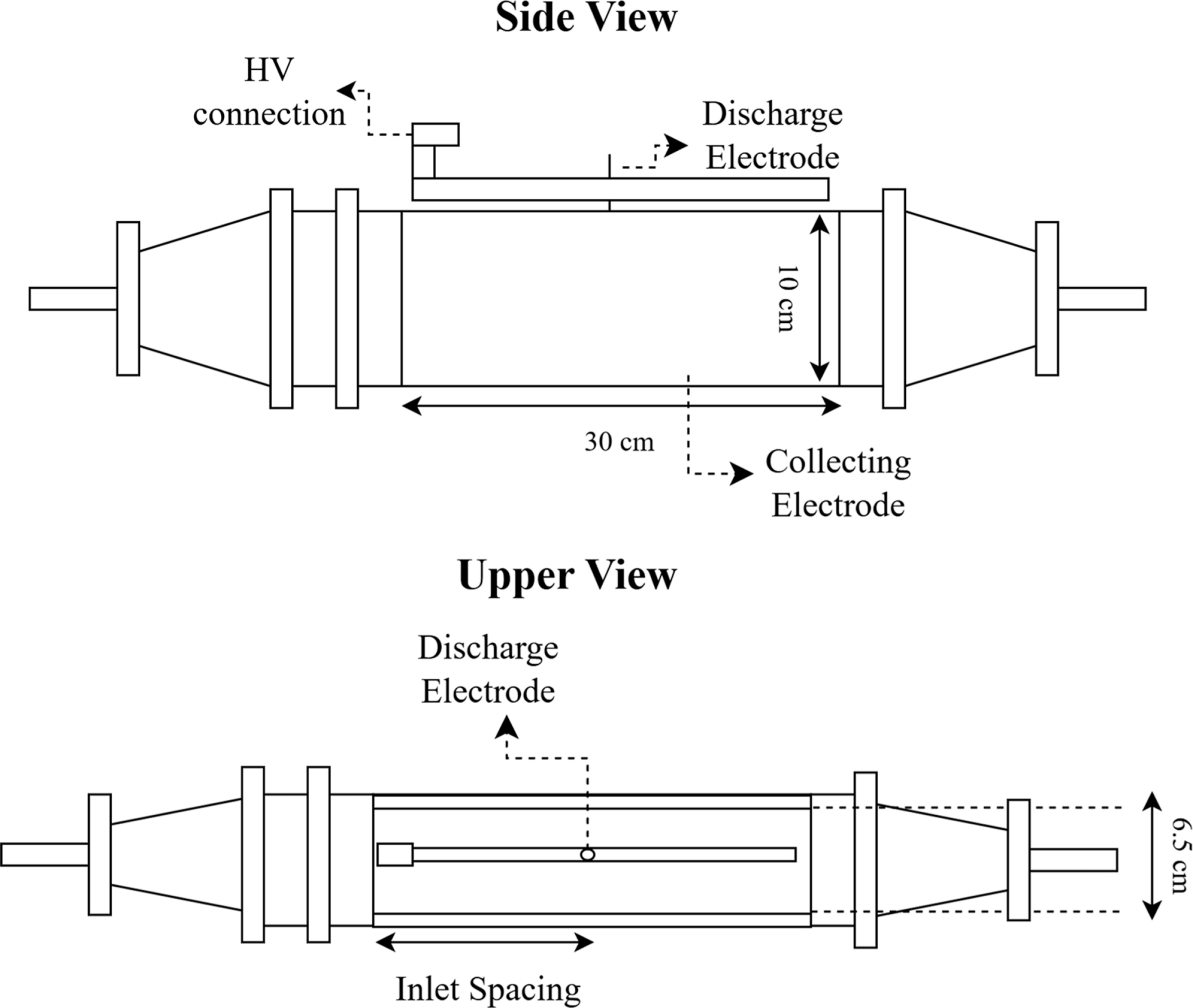
Representation of the side and upper views of the electrostatic precipitator.

Therefore, in the present study, investigation was made of the effect on nanoparticle collection efficiency of the distance between the beginning of the collection plate and the discharge electrode. Figure 3 shows a representation of the position of the discharge electrode for the different spacings, and number of wires used.

**Figure 3:**
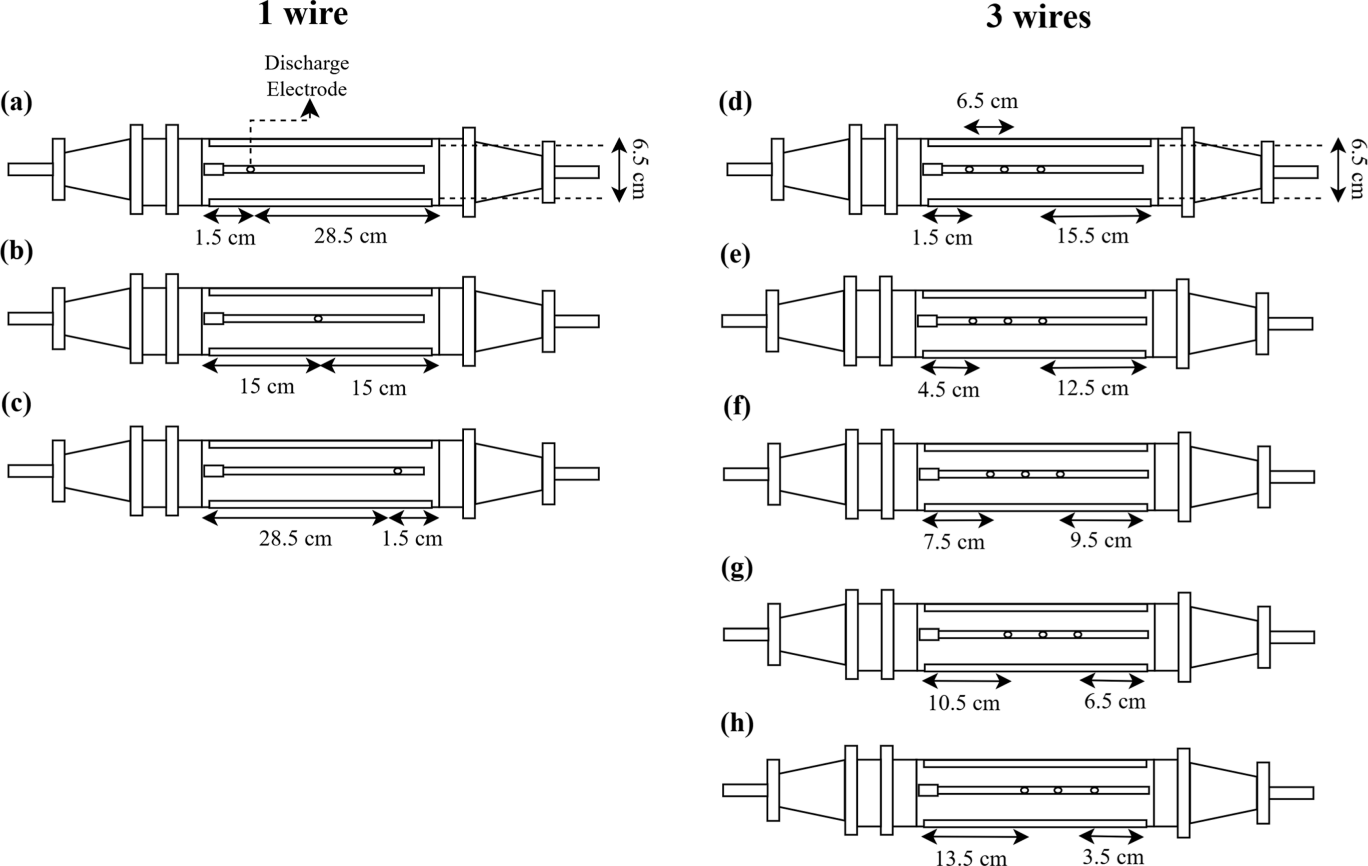
Representation of the inlet spacings evaluated in the electrostatic precipitator (upper view) with 1 discharge electrode, (a) 1.5 cm, (b) 15 cm, and (c) 28.5 cm, and 3 discharges electrodes, (d) 1.5 cm, (e) 4.5 cm, (f) 7.5 cm, (g) 10.5 cm and (h) 13.5 cm.

The inlet spacings were selected to position the discharge electrodes in the initial, central, and final sections of the electrostatic precipitator duct. Hence, for the single wire configuration, the spacings were set at 1.5, 15, and 28.5 cm from the inlet of equipment, while for the 3-wires configuration the spacings were 1.5, 4.5, 7.5, 10.5, and 13.5 cm, as can be seen in Figure 3. These spacings did not consider the distance between the precipitator duct inlet and the start of the collecting plate, or between the end of the collecting plate and the precipitator duct outlet, which were both 2 cm. The inlet spacings were different for both configurations because, in the multiwire configuration, it was necessary to maintain the selected wire spacing of 6.5 cm and in the electrostatic precipitator used there was some limitation regarding the placement of the wires.

For each spacing, experiments were conducted with air velocities of 1, 2, 4, and 5 cm/s. The applied electric field was varied according to the inlet spacing and wire configuration evaluated. The tests were performed in such a way that it was possible to observe the increase in particle collection efficiency until it reached a high value (near or above 90%). However, the change in the location of the discharge electrode and, consequently, in the distribution of the electric field inside the equipment meant that these high efficiencies were achieved with different electric field values.

For the single wire configuration, the spacings of 1.5 and 28.5 cm presented greater similarity and were evaluated using electric fields of 3.08, 3.14, 3.23, 3.29, 3.38, 3.45, 3.48, 3.54, 3.60, 3.69, 3.72, 3.78, and 3.85 kV/cm. For the spacing of 15 cm, electric fields of 3.08, 3.14, 3.23, 3.29, 3.38, 3.45, and 3.48 kV/cm were used. The maximum electric field value applied for each air velocity varied slightly, as required.

On the other hand, experiments with 3-wire configuration were performed with the same electric fields for all inlet spacings, because the multiple wires require a lower electric field to achieve high collection efficiency. Therefore, the values used were 3.08, 3.11, 3.14, 3.17, 3.23, 3.29, 3.35, 3.38, and 3.42 kV/cm.

The particle diameter range considered was between 6.15 and 241.4 nm. A summary of the operational conditions is provided in Table 1. All the experiments were performed in triplicate.

**Table 1:** Experiments Performed

number of wires	inlet spacing (cm)	air velocity (cm/s)	electric field (kV/cm)
1-wire	1.5	1	3.08–3.85
		2	
		4	
		5	
	15.0	1	3.08–3.48
		2	
		4	
		5	
	28.5	1	3.08–3.85
		2	
		4	
		5	
3-wires	1.5; 4.5; 7.5; 10.5; 13.5	1	3.08–3.42
		2	
		4	
		5	

### 2.3 Calculation

The SMPS provided information about the aerosol properties including the median diameter and the geometric standard deviation (σ), as well as the particle concentrations used to calculate the fractional and overall efficiency values, according to eq 1.

1 $$\eta =\frac{C_{i}-C_{o}}{C_{i}}\times 100$$

where, η is the electrostatic precipitator efficiency (%), *C*_i_ is the inlet particle concentration (μg/m^3^), and *C*_o_ is the outlet particle concentration (μg/m^3^). The concentrations used were measured using a particle counter, as described in Section 2.1, that presented an accuracy of ±10%.

In calculations involving the electric field, it is necessary to assume it to be pseudohomogeneous, even though the electric field inside the electrostatic precipitator is not homogeneous. This is due to the difficulty in determining the exact value of the electric field. The value is obtained using eq 2, which relates the applied voltage (*V*) and the distance between the collecting and discharge electrodes (*s*).

2 $$E_{ps}=\frac{V}{s}$$

It is possible to estimate the time (*t*_*n*%_) required for the charging of a particle to reach a value *n* (between 0 and 1), relative to the saturation charge (*Q*_p_^∞^). For this purpose, eq 3 ^11^ was used, where ε_0_ is the electrical permittivity in vacuum (8.86 × 10^–12^ A s/V m), *A* is the surface area of the collecting plates, and *I* is the electric current obtained experimentally (μA).

3 $$\frac{Q_{p}}{Q_{p}^{\infty }}=\frac{t_{n\%}}{t_{n\%}+\left(4\varepsilon_{0}\cdot E_{ps}\cdot A/I\right)}=n$$

The particle saturation charge is described by eq 4,^12^ where ε_r_ is the relative permittivity of the material that constitutes the particle and *d*_p_ is the particle diameter (m). *K*_*n*_ is the Knudsen number (dimensionless) calculated by eq 5, with λ the mean free path between collisions.^12^

4 $$Q_{p}^{\infty }\left(x\right)=\pi \times \varepsilon_{0}\times {d_{p}}^{2}\times |E_{ps}|\times \left[\left(\frac{\varepsilon_{r}-1}{\varepsilon_{r}+2}\right)\times \frac{2}{\left(1+K_{n}\right)}+{\left(1+K_{n}\right)}^{2}\right]$$

5 $$K_{n}=\frac{2\lambda }{d_{p}}$$

The residence time of the particles inside the ESP was calculated as the ratio between the internal volume of the ESP and the flow rate. The electrical migration time and the diffusion time were also calculated, corresponding to the times for the particle to reach the ESP wall by electrical migration and Brownian diffusion, respectively.^41^

Based on the Deutsch equation, the collection efficiency can be calculated using eq 6.^42^

6 $$\eta =1-\exp\left(\frac{-Aw_{E}}{Q}\right)$$

where, *A* is the collecting area (in this case, 0.06 m^2^), *Q* is the air flow (m^3^/s), and *w*_E_ is the migration velocity (m/s). Equation 6 can be rearranged into eq 7 to calculate the migration velocity, as follows^43^

7 $$w_{E}=-\frac{Q}{A}\;\ln\left(1-\eta \right)$$

Since electrostatic precipitation relies on both inertial and electrical forces, discerning the dominant phenomenon involved in particle collection is crucial for understanding the characteristics of the process. This analysis uses calculation of the ratio of the electrohydrodynamic number (*N*_EHD_) and the square of the Reynolds number (*Re* ^2^), obtained using eqs 8 and 9.^44^

8 $$N_{EHD}=\frac{I\times L^{3}}{\rho_{f}\times b\times v_{f}^{2}\times A}$$

9 $$Re=\frac{u\times L}{v_{f}}$$

where, *L* is the characteristic length (in this case, the distance between the collecting electrodes), *b* is the ionic mobility of the ions, *v*_f_ is the kinematic viscosity, and *u* is the gas velocity.

## 3 Results and Discussion

The particle size distribution at the entrance of the electrostatic precipitator is shown on Figure 4 in particle number per cubic centimeter of air sampled (#/cm^3^) as a function of the particle diameter. At an air velocity of 1 cm/s, the total number concentration of particles was 4.29 × 10^5^ and the maximum particle count reached 8.81 × 10^5^ at the particle diameter of 31.1 nm, with the total concentration declining to 3.31× 10^5^ and the maximum to 6.53 × 10^5^ at 28.9 nm and 2 cm/s. This reduction could be attributed to aerosol dilution, since the air velocity increased while maintaining a constant flow rate of 200 L/h in the nanoparticle generator. This pattern persisted at higher air velocities, as can be observed at 4 cm/s, with a total particle concentration of 1.65× 10^5^ and a maximum of 3.32 × 10^5^ at 27.9 nm. In the same way, at 5 cm/s the total concentration was 9.87× 10^4^, while the peak of 2.02 × 10^5^ occurred at 26.9 nm. These values represent both the experiments with 1 and 3 wires.

**Figure 4:**
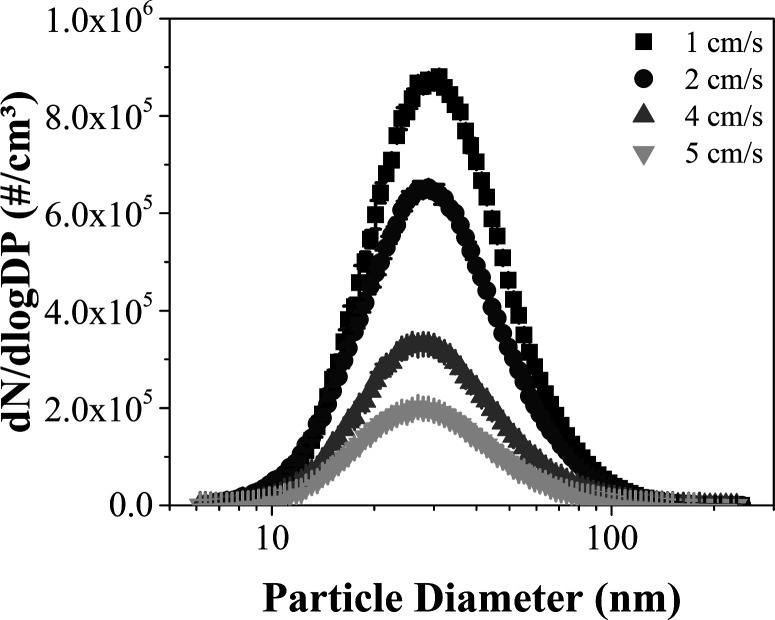
Aerosol granulometric distribution at the inlet of the electrostatic precipitator (numerical basis).

For a better understanding of the data displayed in Figure 4, Table 2 shows the inlet aerosol concentration (from an initial 0.1 g/L NaCl solution), the median particle diameter, the geometric standard deviation (σ) as well as the standard deviation of the triplicate measurements for each value. The aerosol concentration and the air velocity presented inverse behavior, with the aerosol concentration decreasing up to five times as the air velocity increased from 1 to 5 cm/s, according to a nonlinear relationship. The median diameter also decreased with increase of the air velocity, although the difference was not so significant, with the size varying from 30.46 to 28.30 nm. The geometric standard deviation showed no defined pattern according to air velocity, with values in the range from 1.58 to 1.62 nm.

**Table 2:** Inlet Aerosol Concentration (Mass Basis), Median Diameter, and Geometric Standard Deviation (σ) for Each Air Velocity

air velocity (cm/s)	inlet aerosol concentration (μg/m^3^)	median diameter (nm)	σ (nm)
1	45.50 ± 2.55	30.46 ± 0.37	1.58 ± 0.00
2	28.49 ± 2.77	29.33 ± 0.14	1.62 ± 0.01
4	13.15 ± 1.10	28.81 ± 0.09	1.60 ± 0.00
5	8.29 ± 0.38	28.30 ± 0.12	1.60 ± 0.00

Current–voltage curves were obtained for all the operating conditions. However, due to the similar behavior observed across different air velocities and NaCl solution concentrations, this paper presents only the results for an air velocity of 1 cm/s and a NaCl concentration of 0.1 g/L, using 1 and 3 wires with varying spacings (Figure 5).

**Figure 5:**
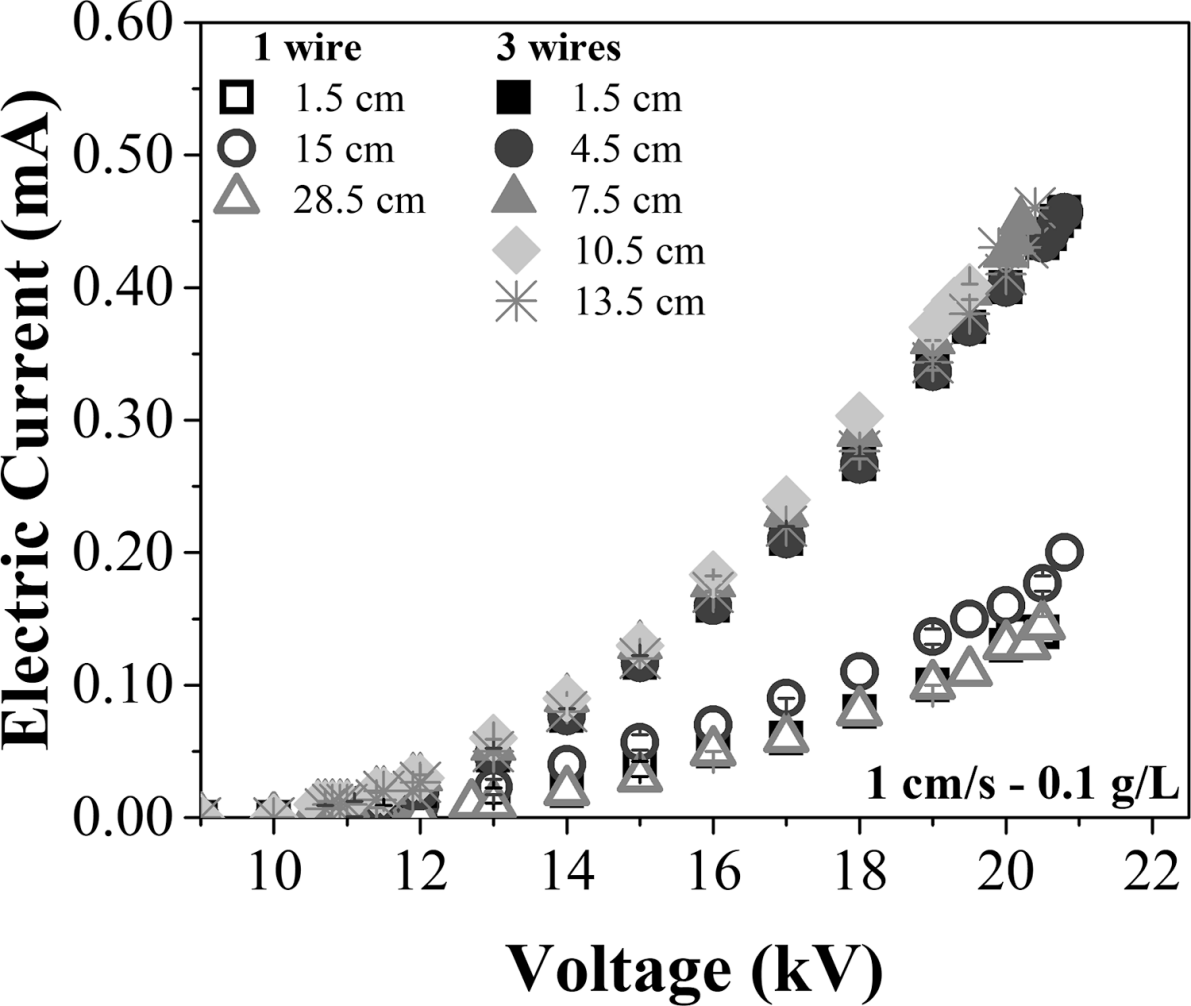
Current–voltage curves with 1 and 3 discharge electrodes, at an air velocity of 1 cm/s, for different inlet spacings.

The curves exhibited very similar behaviors for all the spacings evaluated in both cases. As can be seen in Figure 5, with 1 wire the curves for spacings of 1.5 and 28.5 cm nearly overlapped. However, under all conditions evaluated, the 15 cm spacing resulted in higher electrical current in the voltage range from 13 to 19 kV, indicating a more intense and uniform distribution of the electric field within the electrostatic precipitator when the discharge electrode was positioned at the center of the equipment.^45^

A similar behavior occurred with the 3 wires configuration, as noted by the curves overlapping with different inlet spacings. In this case, the 10.5 cm spacing achieved highest current values, while the 1.5 and 4.5 cm spacings achieved the lowest ones.

As shown in Table 3, the residence times of the particles in the ESP decreased as the air velocity increased. Previous studies associated higher air velocities with lower collection efficiencies, because the possibility of the particles being collected is reduced.^39,40^

**Table 3:** Residence Time for Each Air Velocity

residence time (s)			
1 cm/s	2 cm/s	4 cm/s	5 cm/s
29.18	14.72	7.36	5.88

To better understand the influence of the discharge electrode position on the electrical charging of the particles, the charging times required for the particles to reach saturation charge percentages of 95% and 99% were calculated using eq 6, for the different discharge electrode positions, at 3.85 kV/cm. This electric field was chosen because it was the highest electric field applied in the experiments, to provide stable and comparable values with both configurations. Figure 6 shows a comparison of these charging times with the residence times calculated for different air velocities, with both wires number. It should also be noted that the *y*-axis limit differs in each case, to provide better data visualization.

**Figure 6:**
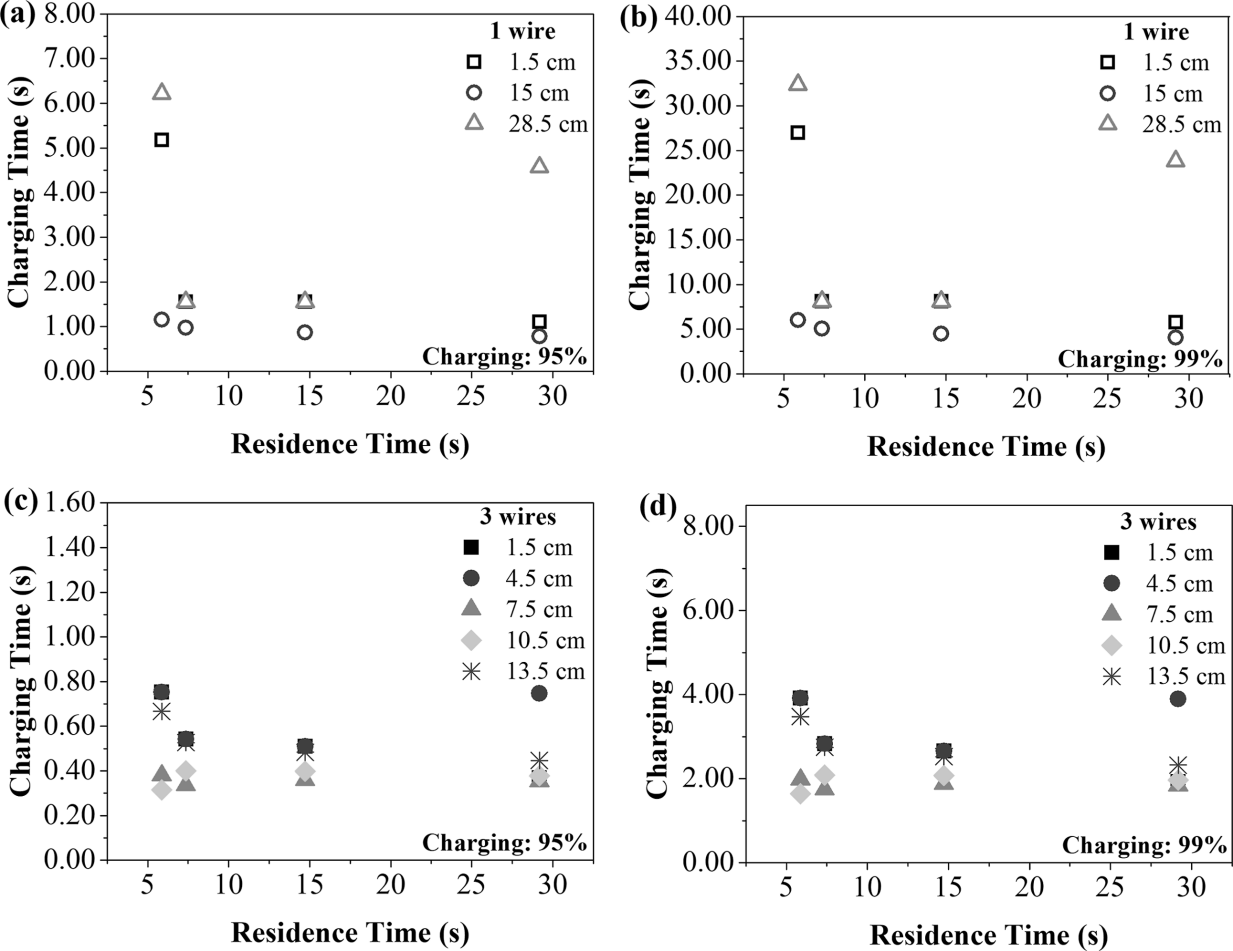
Charging time of the aerosol particles, as a function of residence time for different inlet spacings and charging saturations at 3.85 kV/cm with (a) 1 wire − 95%, (b) 1 wire − 99%, (c) 3 wires − 95% and (d) 3 wires − 99%.

With 1 wire (Figure 6a,b), positioning the discharge electrode at the center of the ESP (15 cm) resulted in the shortest particle charging times, as predicted by eq 6. These times were consistently lower than the residence times and exhibited minimal variations for the different conditions evaluated. These small differences could be indicative of more uniform electrical charging, resulting from the location of the discharge electrode in the center of the electrostatic precipitator, even when different air velocities (and, consequently, residence times) were used. Although the curves behavior is almost the same, the charging time to achieve 99% charging (Figure 6b) was around 5 times longer when compared to 95% (Figure 6a). Furthermore, the short charging times indicated that this configuration could provide more efficient electrical charging and improved particle collection efficiency. Considering the configuration with 3 wires, to achieve an electrical charge of 95% (Figure 6c,d), the charging time required reduced in almost 9 times for all spacings. For both percentages, the charging times still maintained values lower than the residence times used, with the longest time of 3.92 s.

Molchanov et al.^7^ evaluated the Nt-product, that is the product of number of ions concentration and the charging time. The authors observed that an increase of this product above 4 × 10^14^ ions × s/m^3^ (removal efficiency around 93%) has no impact on particles below 50 nm and it is considered hard to obtain. In addition, they also suggest that increasing the electric field strength consistently enhances particle collection efficiency. According to Parker,^12^ under normal electrical conditions, particles will reach 90% of their saturation charge in 10 ms. Sung et al.^45^ suggest that a charging time in the order of ms indicate the dominance of field charging, while longer charging times may be associated with the influence of diffusion charging.

Although the charging mechanism may present a certain influence of the diffusion charging, associated with the low air velocities used in the experimental conditions of this study, the field charging is assumed as the most significant one. This interpretation is due to the values of the dimensionless number *N*_EHD_/*Re* ^*2*^, calculated using eqs 7 and 8, and are greater than 1 for all the conditions, in a wide range that goes from 2 to around 2500 depending on the operating conditions. These results suggest that the electric field exerted a significant influence on the flow pattern and particle trajectories within the ESP, irrespective of the gas velocity. In plate-wire type electrostatic precipitators the ionic wind strongly influences the primary flow when the electrohydrodynamic flow values are much higher than 1. This is because the ionic flow generated by the electric field disturbs the primary gas flow, due to the formation of local vortices, increasing turbulence near the wires.^28,29^ The results obtained here also indicate that the location of the discharge electrode inside the ESP influenced the electrohydrodynamic behavior of the equipment, with the location of the electrode closer to the inlet or outlet resulting in lower current–voltage values (Figure 5) and longer charging times for the particles to reach saturation (Figure 6). Zhu et al.^46^ observed that the diffusion charging had an impact after the insertion of particles from 300 nm to 1 μm, at 2.5 m/s. Besides that, the field charging was the most significant mechanism when these particles were closer to the electrode or had a larger size.

The influence of the electrode location can also be seen in the overall nanoparticle collection efficiency as a function of the electric field inlet spacing, for each wire configuration and air velocity (Figures 7 and 8). In the case of 1 wire (Figure 7), the overall collection efficiencies for the different discharge electrode positions showed that locating the wire exactly in the middle of the ESP duct resulted in the highest collection efficiency performance, with lower electric fields.

**Figure 7:**
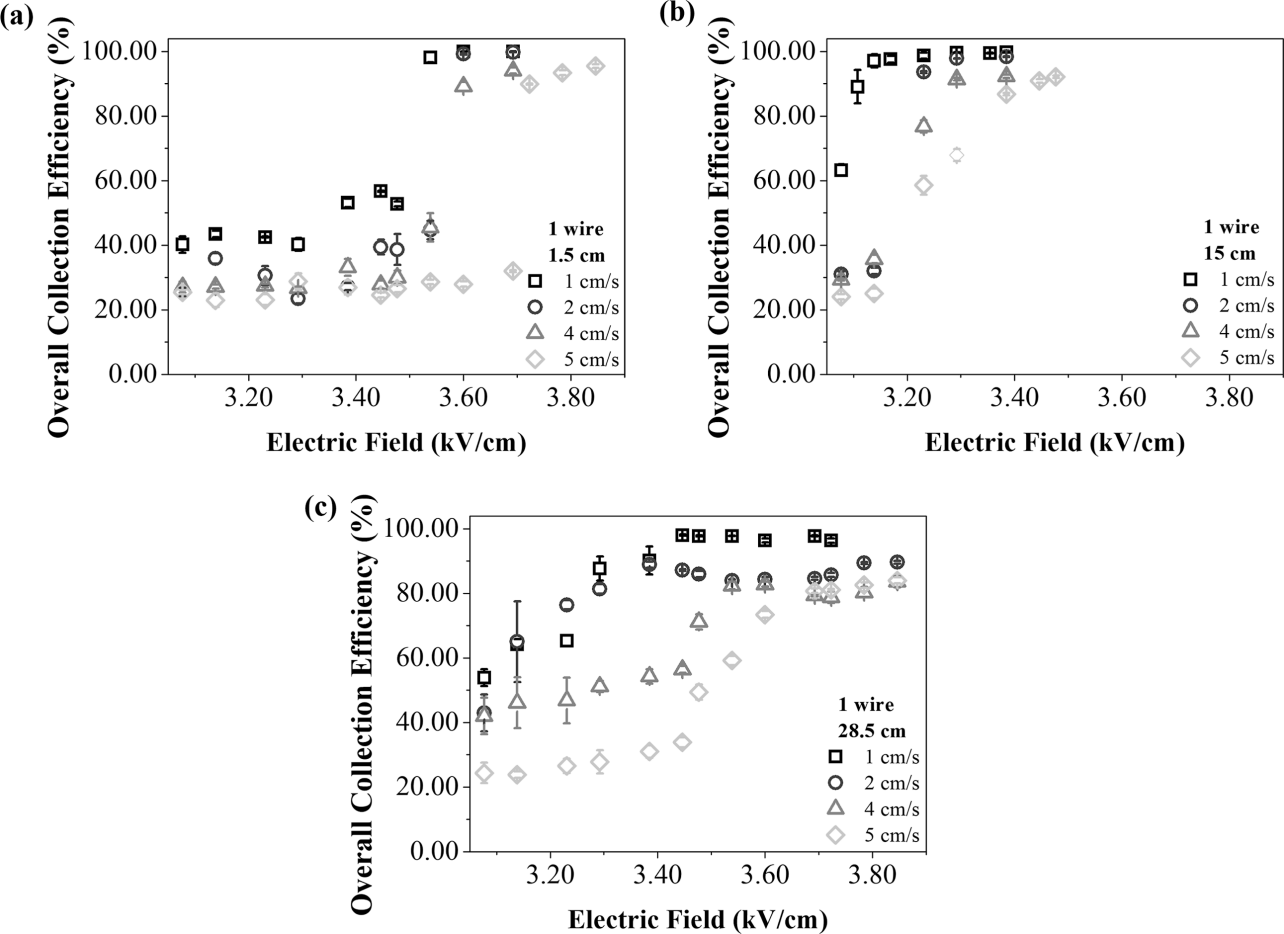
Overall collection efficiencies as a function of the electric field, with 1 wire, different air velocities and the inlet spacings of (a) 1.5 cm, (b) 15 cm and (c) 28.5 cm.

**Figure 8:**
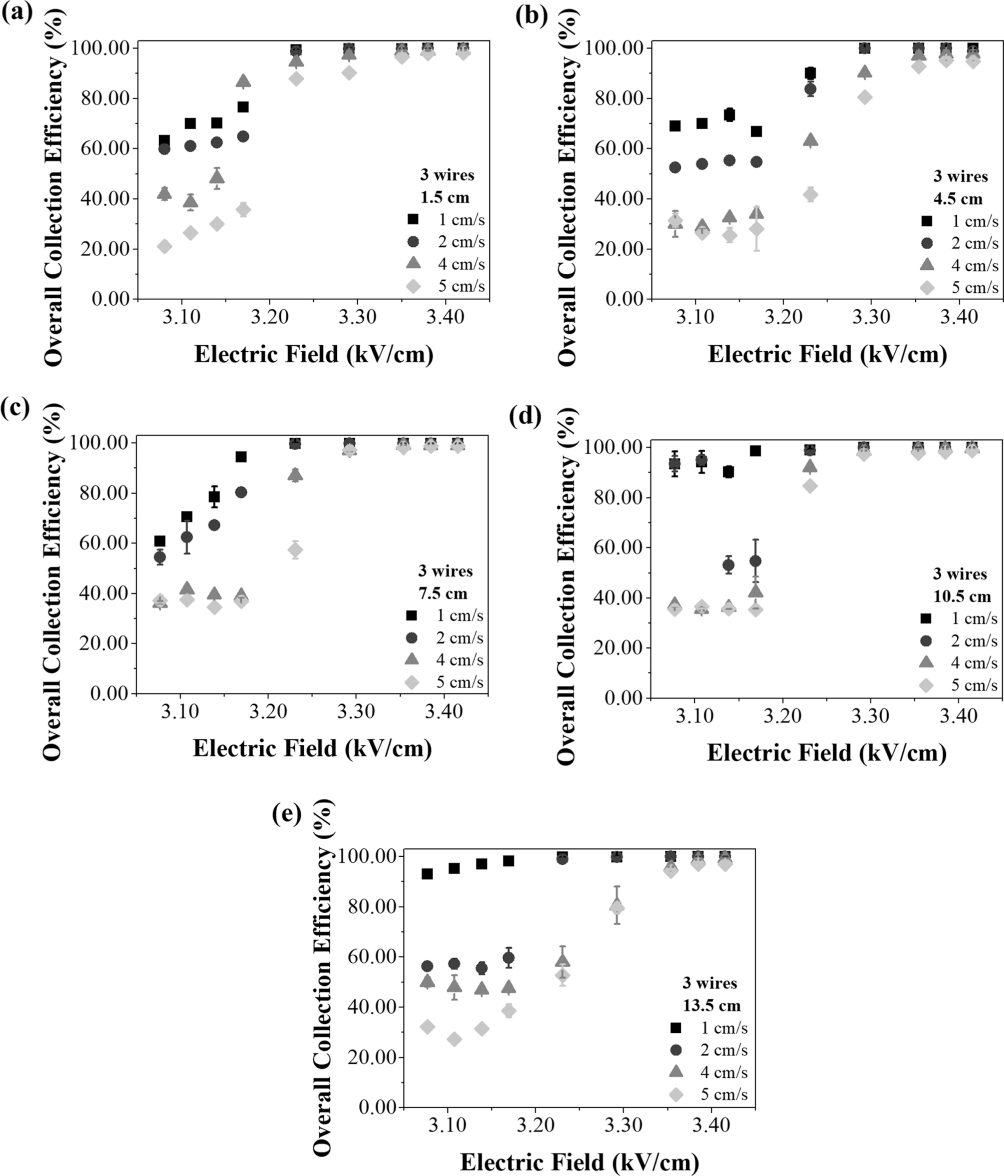
Overall collection efficiencies as a function of the electric field, with 3 wires, different air velocities and the inlet spacings of (a) 1.5 cm, (b) 4.5 cm, (c) 7.5 cm, (d) 10.5 cm and (e) 13.5 cm.

The 15 cm (Figure 7b) spacing probably provided a more uniform and symmetrical distribution of the electric field along the length of the collecting electrode, resulting in maximum collection efficiencies of between 92% and 99.7%, with electric fields of around 3.38 to 3.48 kV/cm, at all air velocities. For the inlet spacings of 1.5 (Figure 7a) and 28.5 cm (Figure 7c), the highest efficiencies were obtained using applied electric fields of up to 3.85 kV/cm. Nonetheless, sharp increases in efficiency were observed, with differences of 20–50% for a small increase in the applied electric field. The increase in efficiency was more sudden with a spacing of 1.5 cm, likely because the combination of a low electric field and the wire positioning in the initial region of the ESP did not favor particle collection. As the electric field increased, it contributed to improved collection efficiency. For spacings of 15 cm (Figure 7b) and 28.5 cm (Figure 7c), the increase was more gradual, as the wire placement was more favorable for particle collection even at lower electric fields.

With 3 wires (Figure 8), the effect of air velocity showed the expected behavior, i.e. a reduction in particle collection efficiency as the air velocity used increased, except for a few conditions. The first of these were with an inlet spacing of 1.5 cm (Figure 8a), an air velocity of 4 cm/s and an electric field of 3.17 kV/cm. In this condition, it was expected that particle collection efficiency would be lower than that obtained at 1 and 2 cm/s, contrary to the result obtained. The other points were observed with an inlet spacing of 10.5 cm and an air velocity of 2 cm/s (Figure 8d). This curve shows a sharp decline at the points of 3.14 and 3.17 kV/cm. In this case, it was expected that these points would show high efficiency, as observed with the other electric fields at this velocity, or that the initial points of 3.08 and 3.11 kV/cm would show lower efficiencies, following the profile of the air velocities of 4 and 5 cm/s.

These points of lower efficiency may indicate an effect of electrical shielding in these conditions^47^ or the initial points of high efficiency occurred because of the lower air velocity combined with the distribution of the electric field with the 10.5 cm spacing. It is interesting to see how the behavior of the curves at 1 and 2 cm/s changed with the different inlet spacings, especially up to the electric field of 3.23 kV/cm, with the greatest difference between the two velocities (around 30%) at the 13.5 cm spacing (Figure 8e).

Pan et al.^48^ observed that the particle concentration was higher near the central region of a dry ESP, with little variation along the duct. Moreover, the particle collection was calculated according to the balance of forces acting in the ESP and it was observed that those forces change according to the region because they are affected by the electric charge and the velocity gradient. In this study, the particle collection was increased alongside the *x*-direction (airflow direction), due to the use of a heat exchange system along the ESP duct.

The differences between the overall collection efficiency with 1 and 3 wires at the lowest and highest air velocities, with the inlet spacing of 1.5 cm, are exhibited on Figure 9.

**Figure 9:**
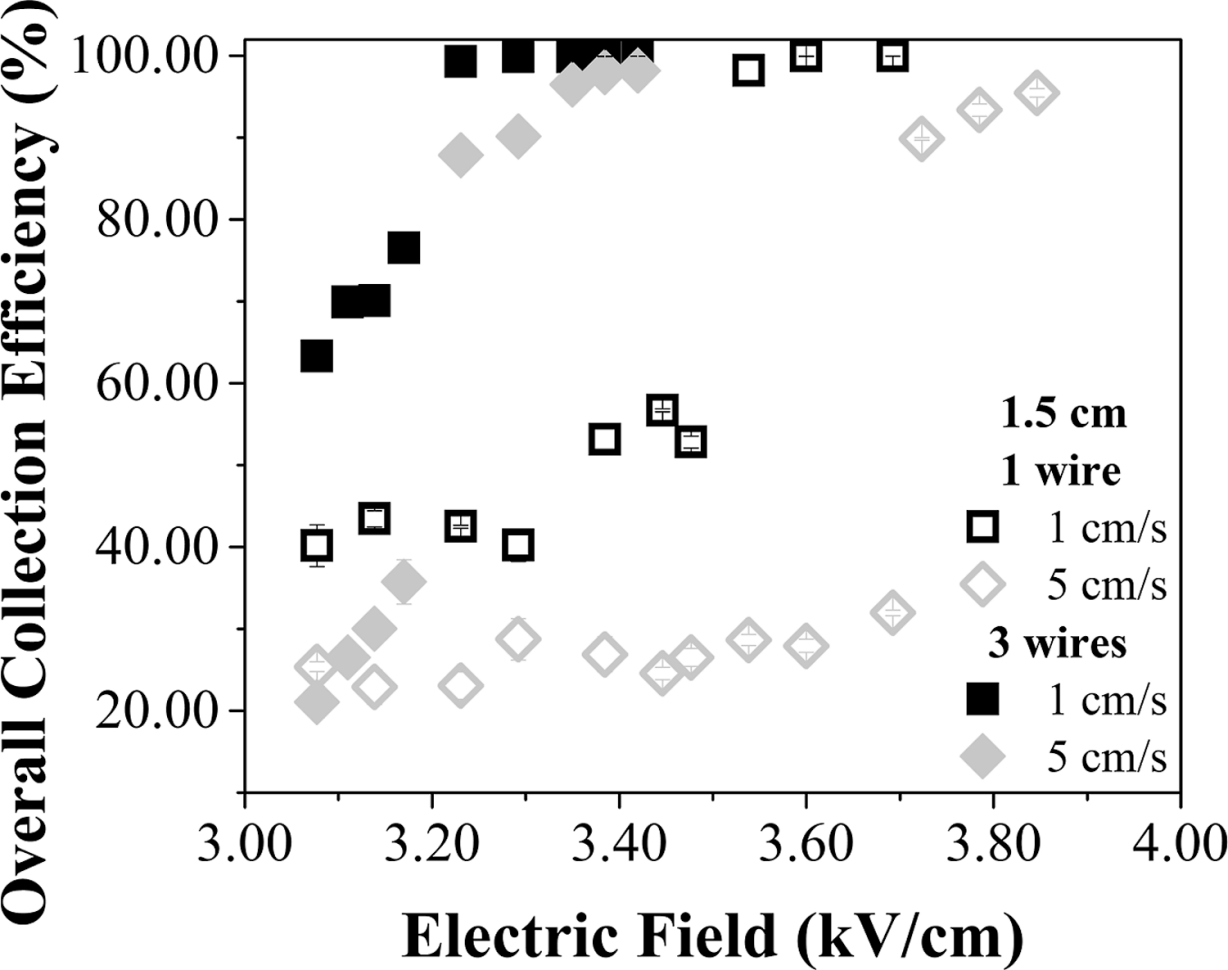
Comparison between the overall collection efficiencies as a function of the electric field, with 1 and 3 wires at the air velocities of 1 and 5 cm/s, with the inlet spacing of 1.5 cm.

As expected, the particle collection was higher when using 3 wires, due to higher electric currents. With both configurations, at the air velocity of 1 cm/s the particle collection was more efficient because there was a predominance of electrical effects in the flow pattern, as well as longer residence times of the particles in the ESP. In the case of 1 wire, the ESP collection efficiency was around 20% higher at 1 cm/s compared to 5 cm/s across most of the electric field range. However, this difference increased up to 80% between 3.45 and 3.48 kV/cm. Besides that, under certain conditions, high overall efficiency values were obtained, even when operating the equipment with only one discharge wire and at low air velocities. With 3 wires, this gap was more evident with the electric fields until 3.20 kV/cm, with differences around 40%. However, these conditions of maximum efficiency varied significantly, depending on the position of the discharge electrode inside the equipment. Better performance was observed when the electrode was positioned in the central region, due to the more favorable electric field distribution in that position. As showed by Fayyad, Asipuela and Iváncsy^49^ the electric field strength significantly decreases in the region between two discharge electrodes, but increasing the number of wires and reducing their spacing results in less variation in the electric field strength. Therefore, with the wires placed in the central position, this region of the ESP duct will exhibit a higher electric field strength.

In the case where the discharge electrode was positioned in the initial region of the ESP duct, closer to the start of the collecting plate, the particles passed through the region of greatest electric field intensity with a faster migration velocity^46^ immediately after entering the ESP. Consequently, most of the particles must have been collected on the initial part of the collecting plate. However, there was a region inside the electrostatic precipitator duct where the electric field intensity was lower, due to a region without any discharge wires, so some of the particles could have lost the acquired electrical charge and were not collected. Particles with low electrical resistivity, such as those composed of NaCl, exhibit faster discharging.^47^ Wang et al.^27^ reported that the numerical electric field distribution in an ESP with a spiked electrode was highest near the discharge electrode, followed by a sharp decline and then a gradual decrease toward the collecting electrode. A similar pattern likely occurred in the regions without wires of the ESP.

These results can be analyzed by comparing the initial, central, or final regions of the ESP. The initial region corresponds to inlet spacings of 1.5 cm for the single-wire setup and 1.5 and 4.5 cm for the three-wire setup. In the central region, the wires were positioned with an inlet spacing of 15 cm for the single-wire setup, and 7.5 and 10.5 cm for the three-wire setup. Finally, for the final region, an inlet spacing of 28.5 cm was used with one wire, and 13.5 cm with three wires. The inlet spacings vary between the two wire configurations due to the arrangement of additional wires in the multiwire setup. Comparison of the inlet spacings in the initial and final region showed that under a low electric field, the collection efficiencies were higher when the discharge wire was positioned in the final region of the ESP. On the other hand, for the highest electric field assessed, the collection efficiency was highest for the wires in the initial region. For the location at the final region, the particles entered the ESP and traversed this distance in a region of low electric field intensity. Hence, particle collection was favored at the lowest air velocity (Figure 7a), since the longer residence time favored charge acquisition in the region of lower electric field. Instead of losing charge in the final region of the electrostatic precipitator duct, the charge was increased, as the particles passed through the region of greatest electric field intensity before leaving the equipment. This can be associated with the greater electrostatic force that acts on particles near the discharge wires.^17^ However, under some conditions, the maximum collection efficiencies achieved with the wire in the final region were lower than those obtained with the initial and central regions. The increase in collection efficiency with higher electric fields is associated with greater ionization of the air and a stronger Coulomb force on charged particles, resulting in faster movement of the particles toward the collecting plates,^49^ consequently increasing the probability of collection.^45^ However, an adequate residence time in the equipment is required for the electrical charging and collection of particles. Therefore, the location of the discharge electrode close to the outlet of the equipment was unfavorable for particle collection at higher air velocities, even under high electric fields, compared to the other spacings evaluated.

Although the Deutsch model is a pioneering and important model for predicting the efficiency of electrostatic precipitators, it includes simplifications that limit its use, such as the assumption of a uniform aerosol distribution in the gas stream and the neglect of particle re-entry. Furthermore, the present study evaluated the collection of nanoparticles in an ESP operating at low air velocities, favoring the occurrence of ionic wind. Another important parameter was the size of the particles, which could have been associated with increased electrical charging by diffusion and other phenomena. These are aspects that the Deutsch model is unable to address, due to its simplicity. However, this model can be used to obtain effective migration velocity data that can be valuable for the design and study of electrostatic precipitators at different scales. Therefore, the effective migration velocity was calculated using eq 3, applying the overall collection efficiencies obtained experimentally (described in Subsection [Sec sec2.3]). The migration velocities for each wire configuration, inlet spacing, and air velocity are shown in Figure 10. In this case, the migration velocities are presented in units of cm/s, to facilitate comparison with the air velocity. With 1-wire, the inlet spacing of 1.5 cm presented the lowest migration velocities for all the air velocities evaluated, followed by the inlet spacing of 28.5 cm, while the highest values were obtained for the 15 cm spacing. On the other hand, the increase of migration velocity with the 3-wires configuration did not present a relation as evident as with 1-wire, but they achieved higher migration velocities in most cases. The migration velocity in the ESP was also influenced by the applied voltage, which affected the strength of the electric field.^42,50^ In most cases, a higher applied electric field led to a corresponding increase of the migration velocity, although this did not occur under some conditions. This could be explained by the lower drag and electric forces in the case of nanoparticles.^43^

**Figure 10:**
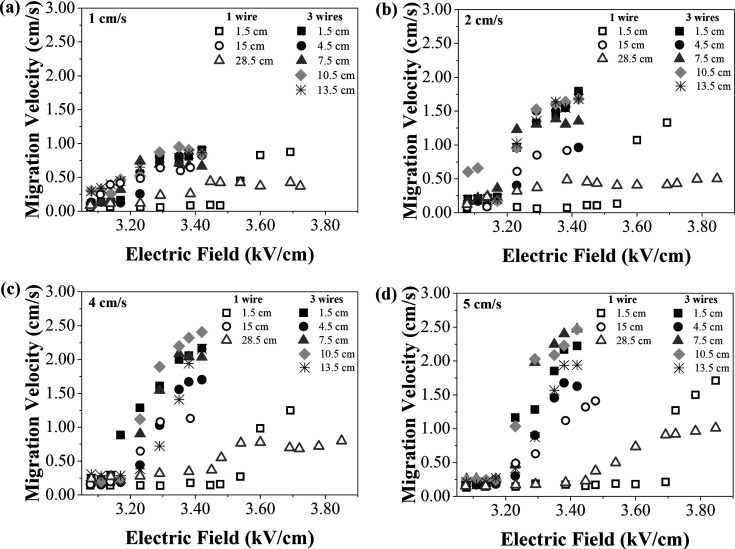
Migration velocities for different inlet spacings and air velocities of (a) 1 cm/s, (b) 2 cm/s, (c) 4 cm/s, and (d) 5 cm/s.

Gao et al.^51^ conducted a numerical investigation into particle migration under various electrode configurations. The study employed operating conditions of an air velocity of 1 m/s, a voltage of 60 kV and a corresponding electric field of 3 kV/cm, which closely aligns with the range of 3.08–3.85 kV/cm applied in the present work. For particles in the size range of 50–100 nm, the study reported migration velocities decreasing from 15 to 7 cm/s. While these values are greater than those achieved with 1 and 3 wires (Figure 10) they remain within the same order of magnitude. Given the similarity in electric field strength between the two studies, the elevated migration velocities observed in the literature are likely attributable to the higher air velocities used in their simulations.

Although the overall efficiency provides important information about ESP performance, fractional efficiency shows how collection is affected by the particle diameter. Figure 11 presents the fractional efficiency values obtained with electric fields of 3.14 and 3.38 kV/cm, which were selected to illustrate the influence of this parameter on the results, for all the air velocities tested.

**Figure 11:**
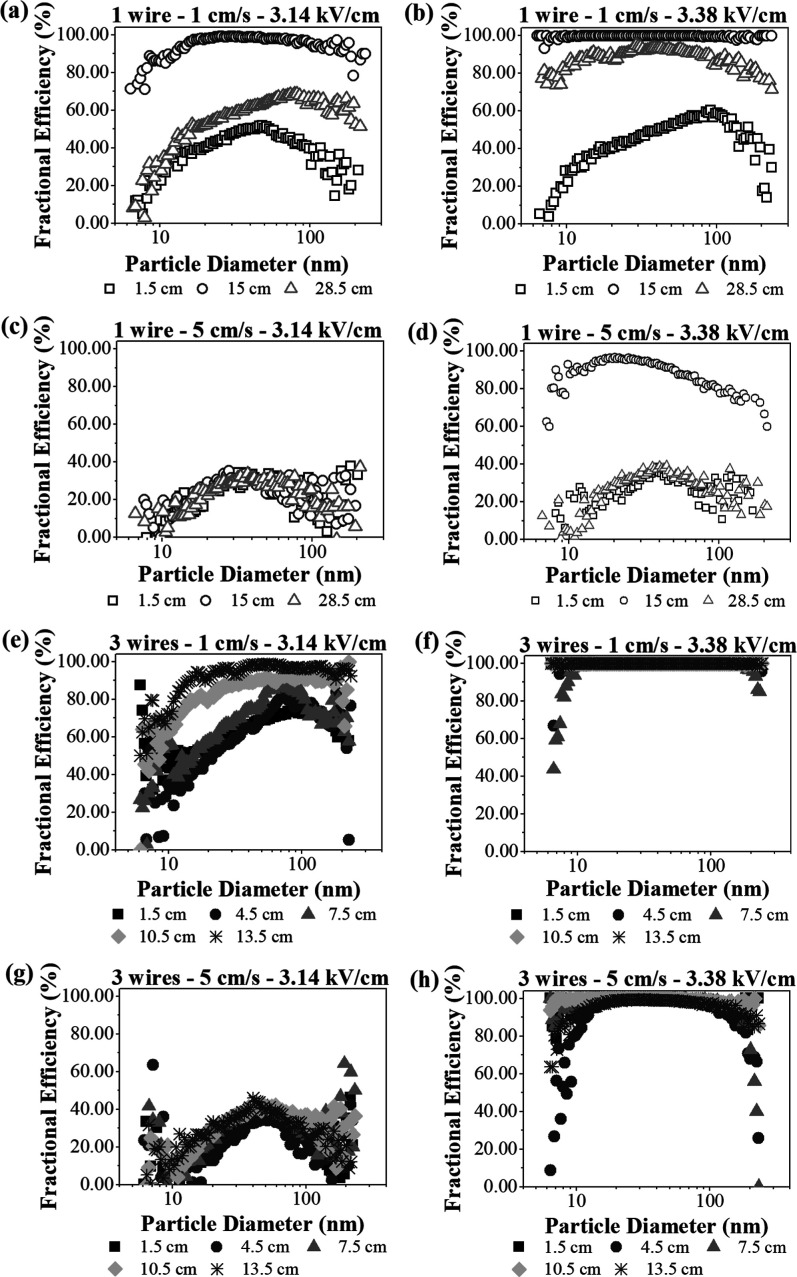
Fractional collection efficiencies for different inlet spacings, with air velocities and electric fields of (a) 1 cm/s and 3.14 kV/cm, (b) 1 cm/s and 3.38 kV/cm, (c) 2 cm/s and 3.14 kV/cm, (d) 2 cm/s and 3.38 kV/cm, (e) 4 cm/s and 3.14 kV/cm, (f) 4 cm/s and 3.38 kV/cm, (g) 5 cm/s and 3.14 kV/cm, and (h) 5 cm/s and 3.38 kV/cm.

Under some ESP operational conditions, particle collection was less efficient, so it was possible to identify a peak of maximum efficiency and the corresponding particle diameter. It can be seen from Figure 11 that the experiments performed with an electric field of 3.38 kV/cm resulted in higher particle removal in specific cases.

With the 1-wire configuration (Figure 11a–d), at the air velocity of 1 cm/s, the maximum efficiencies for the inlet spacing of 1.5 cm were 51.95% at 3.14 kV/cm (Figure 11a) and 59.15% at 3.38 kV/cm (Figure 11b), with the corresponding diameters increasing from 47.8 to 101.8 nm, respectively. At 3.14 kV/cm (Figure 11a), the inlet spacing of 28.5 cm resulted in a peak removal of 68.87%, with a particle diameter of 82 nm. When the applied electric field was increased to 3.38 kV/cm, the maximum point was less evident. For both conditions, the collection efficiencies with the inlet spacing of 15 cm were higher than 90%, confirming the good performance of this configuration. These decreases in collection efficiency were also observed in the work of Badami et al.^52^ on a wet electrostatic precipitator, where minimum values occurred with particle sizes from 70 to 100 nm and were associated with lower electrical mobility.

When the air velocity was increased to 5 cm/s it resulted in lower collection efficiency. At an electric field of 3.14 kV/cm (Figure 11c), the values were almost the same for all the inlet spacings, ranging from 33.69% to 35.52%, with the corresponding particle diameters ranging from 27.9 to 37.2 nm. At 3.38 kV/cm (Figure 11d), the peak removal efficiency with the inlet spacing of 15 cm was again higher than 90%, showing that locating the discharge wire in the central region of the equipment could provide good performance, even at an air velocity of 5 cm/s. Nevertheless, at 3.38 kV/cm, the peak removal efficiency for the inlet spacings of 1.5 and 28.5 cm were around 38.42% and 39.13%, respectively, corresponding to particle diameters of 28.5 and 38.5 nm.

On the other hand, for the 3-wires configuration (Figure 11e–h), at a velocity of 1 cm/s, it can be easily seen that the inlet spacings of 10.5 and 13.5 cm achieved the highest particle collection efficiencies in all the electrical fields evaluated. In addition, they did not have a well-defined optimum operating range, but in some cases (Figure 11e) higher efficiency was observed between the particle diameters of 30 and 80 nm. Increasing the electric field to 3.38 kV/cm resulted in collection efficiencies above 99% for most of the range evaluated.

At an air velocity of 5 cm/s, with the electric field 3.14 kV/cm (Figure 11g), the particle collection efficiencies were close with all the spacings evaluated, with maximum values of around 50% efficiency for particles between 40 and 50 nm. Similarly to the air velocity of 1 cm/s, the electric field of 3.38 kV/cm (Figure 11h) also increased the collection efficiency for all the inlet spacings, with values above 90% in the range evaluated.

Most of the curves exhibited scattered points in their final regions for particle diameters exceeding 100 nm, a behavior attributed by De Aquino Lima and Guerra^53^ to the limited number of particles within this size range. As shown in Figure 4, the median diameter of the aerosol ranged from 28.30 to 30.46 nm.

The fractional efficiency results mostly showed low removal of particles in the size range from 10 to 40 nm, extending to a higher diameter range, depending on the operating conditions. This could be explained by the weaker electrostatic and drag forces for small particles, which increases their residence time and reduces the velocity of migration to the collector plates.^54^ The increase of the electric field to 3.38 kV/cm was not sufficient for the ESP to achieve high collection efficiencies in the entire particle size range for the inlet spacings of 1.5 and 28.5 cm. This suggested that at higher air velocities, increasing the electric field has a limited effect in improving electrostatic precipitator performance.^24^

From the results presented, it is clear the influence of using 1 or 3 discharge electrodes. The results with 1 discharge electrode showed a more coherent behavior, because by using only 1 wire, some secondary effects are eliminated or reduced (such as electrical shielding). In addition, this configuration allowed for a clearer analysis of the variation in collection efficiency as the input spacing changed, as the electric field influence observed in the multiwire configuration did not occur. On the other hand, with 1 discharge electrode, lower electric currents were achieved and, consequently, higher electric field values were required to obtain high collection efficiency. This was especially true for the 1.5 and 28.5 cm spacings.

In the case of the 3-electrode discharge configuration, the electrostatic precipitator performed more efficiently overall, due to the higher electric current and shorter charging time required to achieve high charging percentages. For both cases, the input spacings that promoted the best and most stable results were those in which the electrodes were distributed in the central region of the electrostatic precipitator. In addition, the collection efficiencies achieved with 1 discharge electrode and with the 15 cm inlet spacing were on a par with those obtained with 3 discharge electrodes.

## 4 Conclusions

The performance of an electrostatic precipitator is directly influenced by the operational conditions and the geometric characteristics of the device. The present work evaluated the effect on particle collection efficiency of varying the inlet spacing, considered as the distance from the inlet of the electrostatic precipitator duct to the first discharge electrode, using a single discharge electrode and different air velocities and electric fields. In the 1-wire configuration, the 1.5 cm inlet spacing had the lowest performance due to poor charging and higher particle discharge, although high electric fields still achieved good efficiencies. The 15 cm spacing yielded the highest efficiency due to uniform particle charging and collection without needing high electric fields. At 28.5 cm spacing, reasonable efficiency was achieved with air velocities of 1–2 cm/s, while velocities of 4–5 cm/s required electric fields over 3.54 kV/cm. In the 3-wires configuration, no direct relationship existed between inlet spacing and efficiency, as each spacing had optimal conditions based on air velocity and electric field. The 7.5 and 10.5 cm spacings performed well across most conditions, while 1.5 and 13.5 cm achieved high efficiencies with electric fields above 3.23 kV/cm. The 4.5 cm spacing showed the lowest efficiency overall, despite similar current–voltage behavior to the 1.5 cm spacing, underscoring the impact of spacing and particle charging time on efficiency. Additionally, higher air velocity and reduced residence time impacted efficiency across all spacings. These results allow the study of the geometry of the electrostatic precipitator to be approached from a new perspective, with the evaluation of new parameters and configurations.
